# Two Thalamic Regions Screened Using Laser Capture Microdissection with Whole Human Genome Microarray in Schizophrenia Postmortem Samples

**DOI:** 10.1155/2020/5176834

**Published:** 2020-05-31

**Authors:** Kalindi Bakshi, Eileen M. Kemether

**Affiliations:** Department of Psychiatry, Icahn School of Medicine at Mount Sinai, One Gustave L. Levy Place, NYC, NY 10029, USA

## Abstract

We used whole human genome microarray screening of highly enriched neuronal populations from two thalamic regions in postmortem samples from subjects with schizophrenia and controls to identify brain region-specific gene expression changes and possible transcriptional targets. The thalamic anterior nucleus is reciprocally connected to anterior cingulate, a schizophrenia-affected cortical region, and is also thought to be schizophrenia affected; the other thalamic region is not. Using two regions in the same subject to identify disease-relevant gene expression differences was novel and reduced intersubject heterogeneity of findings. We found gene expression differences related to miRNA-137 and other SZ-associated microRNAs, ELAVL1, BDNF, DISC-1, MECP2 and YWHAG associated findings, synapses, and receptors. Manual curation of our data may support transcription repression.

## 1. Introduction

Schizophrenia (SZ), a complex genetic disorder with no unifying conceptualization of the neuropathological or genetic correlates, is considered “a collection of neurodevelopmental disorders that involve alterations in brain circuits” [1–4]. It is a brain disorder with a heterogeneous symptom profile as well as multiple affected cellular correlates in particular thalamic and cortical circuits [5]. Various functional abnormalities impacting cognition, perception, attention, and affect are evident in persons with SZ [6].

The thalamus is a subcortical brain region comprised of numerous nuclei, many of which have reciprocal connectivity with multiple cortical regions implicated in SZ. Thus, the thalamus is thought of as a nodal link in various neural circuits [7]. As pathology in one brain region can induce both structural and functional abnormalities in either mono- or polysynaptic pathways in other brain regions, we chose to study gene expression differences of a highly enriched neuronal population from a medial tier thalamic nucleus, the anterior (principal) nucleus (AN), that has previously been determined to be a SZ-associated region [5, 8].

Several lines of evidence point to the involvement of the AN in SZ, including deficits of AN volume and neuronal numbers [1, 9–11]. However, these losses were not consistently found [12, 13]. The AN is reciprocally connected with cingulate cortex/paracingulate gyri; efferents of the AN target the hippocampus which then project to mammillary bodies and back to the AN [14–16]. The anterior cingulate cortex was shown to have significant gene expression changes, gray matter, and volume deficits in SZ [17–20]. Functional MRI and FDG-PET neuroimaging studies of SZ subjects have demonstrated relative decreases in blood flow and glucose utilization which has been interpreted as reduced synaptic activity in particular regions of thalamus and cortex leading to a lesser metabolic demand [5]. The thalamus is thus thought to act as a synaptic network, not passively relaying incoming signals, instead integrating hippocampal and mammillary body inputs dynamically in response to preceding neural circuit activity. The AN is considered a nodal link for multiple functional circuits including those subserving motivation, novelty detection, memory, and learning [21, 22]. Synaptic plasticity has been shown to be a major property underlying thalamic function in the adult brain [23, 24]. The AN has been shown to have long-term synaptic modifications and plays an active role in amplifying convergent hippocampal and mammillary body inputs [24, 25]. SZ-relevant changes in discrete thalamic subregions may have an effect in reciprocally connected cortical fields.

The ventral posterior lateral (VPL) nucleus is a lateral tier thalamic nucleus. Its neural circuit is comprised of spinothalamic tract fibers originating at posterolateral medulla going to VPL thalamus and terminating in the primary somatosensory cortex. Neuroimaging has not demonstrated volume loss in the lateral tier nuclei in living subjects with SZ [26]. The VPL, with one report of left hemisphere deficits in SZ [27], is not considered a SZ-associated thalamic subregion [5, 28], and thus was chosen as a control region for AN to study SZ-relevant gene expression defects in the thalamus.

Our aim was to identify brain region-specific gene expression changes and possible transcriptional targets in a disease associated thalamic region which may be involved in the underlying neuropathology of SZ. We used laser capture microdissection to accumulatively collect a highly enriched neuronal population for whole human genome microarray study from two thalamic regions in the same subject for comparison as well as comparison of these two regions in disease (SZ) vs. normal controls (NC). The comparison of a SZ-associated thalamic region (AN) with a largely SZ-unaffected thalamic region (VPL) in the same subject was an effort to minimize the identification of gene expression changes attributed to between-subject heterogeneity and maximize the possibility of identifying disease-specific changes. This is the first study to our knowledge to compare a disease-impacted to a nondisease impacted thalamic region in the same subject with SZ.

## 2. Methods

### 2.1. Materials and Template Preparation

**Figure 1:**
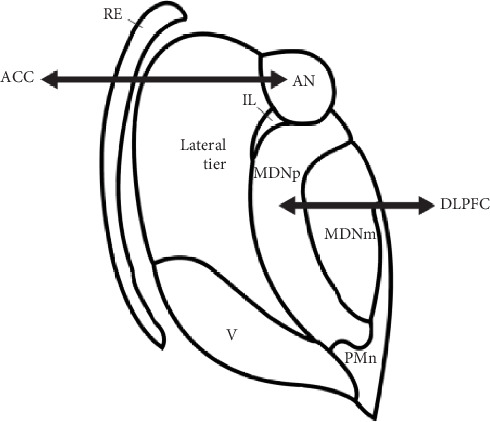
Thalamus drawing with subregions. Anterior (principal) nucleus (AN) reciprocally connected with the anterior cingulate cortex (ACC), mediodorsal nucleus, parvocellular subregion (MDNp) reciprocally connected with dorsolateral prefrontal cortex (DLPFC), and ventroposterior lateral nucleus (VPL), a lateral tier nucleus.

**Figure 2:**
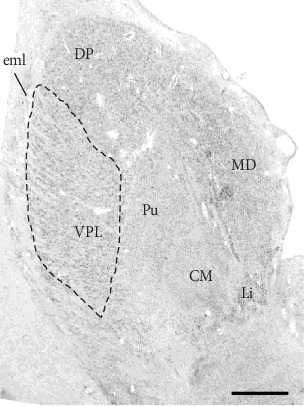
Postmortem Tissue Sample stained for visualization and delineation of thalamus subnuclei. Our delineation of the VPL follows that of the standardized nomenclature of Feremutsch and Simma in Dewulf [105]. We sampled from the portion of VPL in coronal sections through the caudal half of the centromedian nucleus (CM). At these levels, VPL is rather conspicuous due to its relatively larger and more coarsely staining neurons compared to surrounding nuclei. Moreover, these neurons, especially in the more lateral extent of the nucleus exhibit a prominent diagonal arrangement. The lateral boundary of the VPL is the external medullary lamina of the thalamus; its superior border, the dorsal posterior (DP) nucleus with more homogeneously arranged cells; it medial boundary is the anterior portion of the pulvinar with conspicuously smaller, paler, and more homogeneously arranged cells. Abbreviations: CM: centromedian nucleus; DP: dorsal posterior nucleus; eml: external medullary lamina; Li: nucleus limitans; MD: mediodorsal nucleus; Pu: putamen.

Postmortem brain tissue was donated by The Stanley Medical Research Institute's (SMRI) brain collection. All brain specimens were screened (SMRI) to exclude neuropathological abnormalities. The specimens were collected, with informed consent from next-of-kin, by participating medical examiners between January 1995 and June 2002 (SMRI website). The project was not human subject research and thus did not require IRB approval. The cohorts (*n* = 15 each, SZ/NC) were diagnosed using DSM-IV criteria and matched by sex, age, race, postmortem interval (PMI), affected side of the brain, and medication history (Table 1). Researchers were blinded to specimen code until the data set was received at SMRI. Samples were processed (see below) in random order to minimize systematic bias arising from sample preparation. Template preparation was conducted as previously described [29]. Byne et al. delineated VPL and reviewed EK parcellations of both AN/VPL (see [1] (Figure 1 drawing). See photograph of thionin-stained neuron (Figure 2) for the VPL delineation example.

### 2.2. Laser Capture Microdissection, RNA Isolation, and Target Preparation

Tissues were prepared for laser capture microdissection as previously described [29], using biologic grade ethanol. Total RNA was isolated in 10 *μ*l nuclease-free water for each subject using RNeasy Micro Kit (Qiagen, Hilden, Germany) following the manufacturer's instruction. Purified RNA (1 *μ*l) was subjected to quality evaluation by the Agilent Bioanalyzer pico RNA assay (Agilent Technologies, Palo Alto, DA, USA). We visually examined the electropherograms of all RNA samples, and those which showed two peaks of 18S and 28S ribosomal RNA (rRNA) were retained and subjected to amplification and microarray assay regardless of the degree of degradation. The Arcturus PixCell II laser capture microscope system (Mountain View, CA, USA) was used at the following settings: power 40-60 mW, target 0.300 V, temperature 22.4°C, current 4.6 millihertz (mH), repeat-laser pulse time 0.2 s, and duration 650-750 *μ*m. Approximately 3000 neurons, the largest thionin-stained cells, with nucleus and cytoplasm visible, from consecutive sections were individually captured and pooled per subject. Five nanograms (ng) or maximum quantity available of qualified total RNAs were reverse transcribed and amplified using the WT Ovation Pico RNA amplification System (Nugen, San Carlos, CA, USA). The length of the amplified cDNA ranged between 50 and 2000 nucleotides, with peaks of 300-600 nucleotides from randomly sampled cDNAs assayed by Bioanalyzer.

### 2.3. Microarray Hybridization

Five micrograms of the single-stranded cDNAs were fragmented and end-labeled with biotin using FL-Ovation cDNA Biotin Module V2 following the manufacturer's instructions (Nugen, San Carlos, CA, USA). The whole reaction mixture of each sample was made into hybridization cocktail using the HVVS kit (Affymetrix, Santa Clara, CA, USA) following the instruction provided with the Nugen biotin labeling kit and then hybridized to Human Genome U133 Plus 2.0 Arrays (Affymetrix, Santa Clara, CA, USA). The array contains 47 000 transcripts and variants covering 38 500 human genes. The array images were generated through a high-resolution GeneChip Scanner 3000 7G (Affymetrix). The correlated signal intensity of spike-in controls and the percentage of present call generated based on MAS 5.0 within the GeneChip Operating Software (GCOS) were used for data quality control for each array. While our laser capture microdissection targeted neurons specifically, interneurons and incidental concurrent isolation of astrocytic foot plates, corticothalamic synapses on proximal dendrites and cell soma, as well as satellite oligodendrocytes were not completely avoidable (see Figure 3(b). in [30]. Corresponding cell-specific messenger RNAs (mRNAs) contributing to any gene expression changes in this study are considered contaminates and not an accurate reflection of disease-related changes.

### 2.4. Microarray Data Mining and Analyses

The data were divided into disease vs. nonpsychiatric control (schizophrenia (SZ) vs. normal control (NC)) for the disease candidate gene list. From the initial 60 (*n* = 15, each group; two brain regions, AN/VPL), a total of 38 GeneChip data were generated initially. Samples were excluded for various reasons (e.g., not enough RNA isolated, poor RNA quality or hybridization, and poor amplification). The data were analyzed by using R Bioconductor [31] statistic packages in combination with other well-known microarray and pathway analysis tools. Briefly, the raw data were first normalized across the chips by log scale robust multiarray analysis RMA [32] method, and the quality control of each chip data was performed by investigating overall intensity values for all probes, negative and positive controls. The distribution of intensity values across chips was examined, and the outliers were detected. The probe sets with low intensity values or the outliers due to poor RNA quality or hybridization were excluded.

To identify significantly differentially expressed genes between groups, the LIMMA method [33] was used to identify differentially expressed mRNAs for the following comparisons: (1) AN disease vs. AN normal control (AN SZ/NC) (Supplementary Table S1), (2) VPL disease vs. VPL normal control (VPL SZ/NC) (Supplementary Table S2), (3) VPL and AN disease same subject (SZ_VPL vs. AN) (Supplementary Table S3), and (4) VPL and AN normal control same subject NC_VPL vs. AN) (Supplementary Table S4). The gene expression data were also integrated with various types of up to date biological information using Database for Annotation Visualization and Integrated Discovery (DAVID) v6.7 Bioinformatics Resources [34, 35], National Institute of Allergy and Infectious Diseases (NAID), NIH (05/26/2012) http://david.abcc.ncifcrf.gov/, and Ingenuity System (http://ingenuity.com) to perform Gene Ontology (GO) function, pathway and transcription factor binding sites (TFBS) enrichment analysis, and gene/protein association network analysis for better interpretation of gene expression [27] profiles in the context of pathways, biologic function. Ingenuity Pathway Analysis (IPA) is a biological data analysis software program from Ingenuity® Systems which provides biological insight into interactions between genes, pathways, and disease processes in various experimental platforms, including microarray data. Pathway and network analysis of differentially expressed genes was performed (7/13/2015) using IPA (Ingenuity, Redwood City, CA; http://www.ingenuity.com) software licensed by Mount Sinai School of Medicine. The datasets are publicly available for download at https://www.stanleygenomics.org.

## 3. Results

A summary for the total numbers of differentially expressed transcript changes, both up- and downregulated, for each of the conditions is provided in Table 2.

### 3.1. Differentially Expressed Genes in the Anterior Nucleus (AN) of Schizophrenia Brains Compared with Normal Control Brains (AN SZ/NC) Fold Change 2.0, *P* < 0.05

Using the LIMMA method, we compared schizophrenia (*n* = 11) vs. normal controls (*n* = 10) subjects for thalamic AN and identified differentially expressed genes (55 upregulated genes highlighted in red and 482 downregulated genes highlighted in green) listed in Table S1. IPA identified networks, pathways, and upstream regulators are listed in Tables S1.1, S1.2, and S1.3, respectively. Network molecules are related to multiple diseases and functions including hereditary, neurological disease, developmental disorders, and cellular assembly and organization. The top five pathways identified were mitochondrial dysfunction, oxidative phosphorylation, unfolded protein response, protein ubiquitination, and ketogenesis.

### 3.2. Differentially Expressed Genes in the Ventral Posterolateral Nucleus (VPL) of Schizophrenia Brains Compared with Normal Controls (VPL SZ/NC) Fold Change 2.0, *P* < 0.05

Using the LIMMA method, we compared SZ (*n* = 7) vs. NC (*N* = 8) subjects for the thalamic VPL nucleus and identified differentially expressed genes (24 upregulated genes highlighted in red and 22 downregulated genes highlighted in green) listed in Table S2. IPA identified networks, pathways, and upstream regulators are listed in Tables S2.1, S2.2, and S2.3, respectively. Network molecules are related to cell cycle, cell death, and cell-to-cell signaling. Three pathways were identified: BMP signaling, estrogen receptor signaling, and triacylglycerol degradation.

### 3.3. Differentially Expressed Genes in the Same Subject Comparing Two Different Thalamic Regions for Schizophrenia Brain Subjects (SZ_VPL vs. AN) Using Adjusted *P* Value < 0.05

Using the LIMMA method, we compared the two thalamic regions, VPL vs. AN, in the same subjects with SZ (*n* = 8) and identified differentially expressed genes (24 upregulated highlighted in red and 180 downregulated highlighted in green) listed in Table S3. IPA identified networks, pathways, and upstream regulators are listed in Tables S3.1, S3.2, and S3.3, respectively. Network molecules are related to various diseases and functions including tissue morphology, cell-to-cell signaling and interaction, neurological disease, and developmental disorders. Pathways identified included axonal guidance signaling, dopamine-DARPP32 feedback in cAMP signaling, nNOS signaling in neurons, and synaptic long term potentiation. Manual curation of Table S3 identified gene expression changes for 14-3-3 gamma (YWHAG) NCBI interactants **DDX17**, **SPTBN1**, and **SRRM2**; all are downregulated. In addition, manual curation of S3.3 identified MECP2 as an upstream transcription regulator.

### 3.4. Differentially Expressed Genes in the Same Subject Comparing Two Thalamic Regions for Normal Control Brains (NC_VPL vs. AN) Using Adjusted *P* Value < 0.05

Using the LIMMA method, we compared the two thalamic regions, VPL vs. AN, in the same NC subjects (*n* = 9) and identified differentially expressed genes (877 upregulated highlighted in red and 1,153 downregulated highlighted in green) listed in Table S4. IPA identified networks, pathways, and upstream regulators are listed in Tables S4, S4.2, and S4.3, respectively. One hundred ninety-one pathways were identified (Table S4.1).

We provide a comparison of ELAVL1 NCBI identified interactants for NC AN/VPL (Table S4) (133 up- and 113 downregulated) with SZ AN/VPL (Table S3) (22 mRNAs, one up- and 21 downregulated) gene expression changes in Table S9 (see discussion below). Five are overlapping in this comparison.

### 3.5. Manual Curation of our Data Section Supplementary Table 5 (S5): MicroRNA-137 Findings

In our study, microRNA-137 (miR-137 and miR-137-3p) was identified as an activated upstream regulator in (a) our AN_SZ_NC (S1.3) comparison (mir-137 4 target genes; miR-137-3p 48 target genes), (b) miR-137-3p in VPL_SZ_NC (S2.3) comparison (6 target genes) and AN_VPL_NC (S4.3) (dozens of target genes) (S5). MiR-137 was not identified as an upstream regulator in our AN_VPL_SZ comparison (S5). The only predicted activated state was identified in S1.3 (activation *z*-scores of 2.000; 5.280 and *P* value of overlap 1.68*E*-05; 7.28*E*-05). In our S2.3 findings, miR-137-3p was an upstream regulator of ***ADRB1***, ***ASPH***, ***CACNB2*** [36], ***CTNA3*** [37], ***GREM1*** [38], and ***ZEB2*** (an ELAVL1 interactant; see section on ELAVL1 below) (S9). In S3.3 data, miR-137 was not found. In S4.3, dozens of genes were identified as targets of miR-137 upstream regulator.

Supplementary Table 6 (S6): our data compared with [39].

Supplementary Table S7 (S7): our data compared with [40].

Supplementary Table S8 (S8): our data compared with [41]. In the schizophrenia upstream regulator comparisons, all miRNAs were activated (S1.3, S3.3), while in S2.3, of 2 miRNAs represented, neither was activated. In S4.3, of the 6 miRNAs, two were activated (miR-96-5p and miR-134).

Supplementary Table S9 (S9): MeCP2 was identified as an upstream transcription regulator in our SZ comparisons (S1.3, activation z score 0.698, Target molecules in the dataset: GRIN2A, RAB39B, HRASLS, SLC2A3, UBE3A, YWHAB); S3.3, *P* value of overlap 3.86*E*-02, target molecules in dataset APOE, EHMT2, MBP) and control (S4.3 log ratio -0.823; activation -0.239).

ELAV-like protein 1 (ELAVL1; HuR) is one of many MeCP2 interactants (NCBI). Manual curation of our data identified an almost 10-fold difference in gene expression changes for ELAVL1 targets for S3 and S4 reflected in this Supplementary Table S9.

Supplementary Table S10 (S10): manual curation of our data for synapse-related genes [42] and receptors. In S1, there were 6 up- and 12 downregulated genes. In S2, there were 3 up- and 1 downregulated genes. In S3, there were 3 up- and 16 downregulated genes. In S4, there were 43 up- and 92 downregulated genes.

## 4. Discussion

We compared the transcriptome of accumulatively collected neurons from a disease-impacted SZ thalamic nucleus to a nondisease thalamic nucleus in the same subject and each nucleus in SZ vs. NC (S1, S2, S3, and S4). Our discussion will focus on specific genes related to microRNA-137 (S5-7), Ingenuity Pathway Analysis microRNA findings (S8), MeCP2 interactant ELAVL1 target genes from our data (S9), and synapse-related (S10) findings. Gene expression changes identified in our SZ studies will be **bolded** and in our NC studies ***bolded*** and ***italicized***.

A single-nucleotide polymorphism (SNP; rs1625579) in the microRNA-137 gene (MIR137) has been linked with SZ [43, 44]. MicroRNA-137 expression levels were elevated in peripheral blood samples of SZ subjects who were in the initial onset of the disease [45]. Manual curation of our data identified genes related to upstream regulator miR-137 (S5). In S1.3, miR-137 was an upstream regulator for **GABRA1**, **NECAP1**, **NRXN1**, and **SPTLC1** (all downregulated; in S1.3 only). Previous reports of SZ-associated miR-137 gene targets identified in our study are **ATXN1** (up-), **GABRA1**, **GRIN2A** (downregulated) (S1) and ***GRIN2A***, ***NEFL*** (up-) and ***TCF4***, and ***GRM5*** (downregulated) (S4) [46, 47]. SZ-associated gene **ATXN1** [48] is a target of miR-137 [39, 49] (S6). It is also a protein interaction partner of ZNF804A, a target of miR-137, and 18 other SZ genes [50, 51]. Transcriptional or posttranscriptional regulation of the MIR137 gene may be involved in SZ [52]. Alterations in **GABRA1 (**GABA_A_ alpha 1 receptor) subunits have been identified in SZ [49]. **NRXN1** has been linked with SZ [53]. A minor copy number variation in **NRNX1** may impact synapse functioning [54]. Additionally, various target genes of two SZ-associated microRNAs (miR-17-5p, miR-151-3p) found previously to be dysregulated in SZ were identified (Table 3; S3.3) [55]. Other synapse-relevant microRNAs identified in our study are in Table 4 [42]. MiR-132 is necessary for neuronal functioning insofar as neuronal activity regulates its expression [56]. miR-132 was identified as a potential activated upstream regulator of various gene targets in our SZ comparisons (S1.3, S3.3), while in controls (S2.3, S4.3) it was identified but not as an activated regulator. As miR-132 is thought to impact synapses and dendritic complexity [56], our target genes may indicate thalamic synapse- and transcription-related genes in SZ.

MicroRNAs are noncoding RNAs, which can bind to messenger RNA (mRNA) to repress the expression of various gene targets [39]. MicroRNAs have various roles including transcription and translation activation and repression [57]. Each microRNA may have as many as hundreds of target mRNAs [42]. Dysregulation of one microRNA may posttranscriptionally impact the expression of thousands of genes [39]. MicroRNAs may mediate translation repression of mRNAs [41]. Since miRNA:mRNA interactions in the human brain were mapped [46], a window has been opened into the impact of miRNA and mRNA in neuronal functions. miR-137 is highly expressed in various brain regions, and it regulates adult neurogenesis, neuronal maturation, synaptic function, vesicle trafficking, and synaptic plasticity [58–61]. miR-137 is thought to target an AMPA receptor subunit and be necessary for mGluR-mediated synaptic plasticity [62]. Both receptors are implicated in the pathogenesis of SZ. miR-137 targets various mRNAs involved in glutamatergic signaling including regulating proteins in the PI3K-Akt-mTOR pathway which regulate response to both BDNF and neuregulin 1 signaling [41]. miR-137 may also regulate presynaptic vesicles, signals at the synapse, and plasticity of the synapse [41, 59]. As there are multiple signaling pathways and genes in our NC IPA compared to SZ (S7), our microRNA findings may lend support to the possibility of dysregulated transcription which may impact synaptic plasticity or function in the anterior nucleus in SZ.

Evidence for DISC-1 and its interacting partners linked with SZ susceptibility is mounting [63–65]. Our study identified gene expression changes in several DISC-1 targets (**CCDC136**, **GNB1**, **KIF3A**, **PAFAH1B1**, **PGK1**, **SRR**, and **TUBB**) (S1) and **SPTBN1**, **TRIO**, and **UTRN** (S3) [66–68]. Several microRNA upstream regulators were also identified for **KIF3A, PAFAH1B1,** and **TUBB** (S8; S1.3) [41]. DISC-1 target YWHAG was identified as an NMDA receptor-related SZ *de novo* CNV [67, 69]. YWHAG targets of interest in our study are **DDX17** [70], **SPTBN1** [71], **PAFAH1B1** [72, 73], and **SRRM2** [74] (S3.3). **DDX17**, **GNB1**, **PRKCA**, **SRRM2**, and **TUBB** (S9) are also ELAVL1 targets (see next section). Both DISC-1 and ELAVL1 interact with **GNB1** (S1, downregulated). The upregulation of GNB1 was identified in the anterior cingulate cortex (ACC) of control subjects [75]. Abnormalities of DISC-1 protein may impact various proteins involved in pathways, which are vital to synapse formation, development, and maturation, including, but not limited to, glutamatergic, AMPA, and NMDA receptor complexes [66]. Our findings lend support to the dysregulation of DISC-1 and/or YWHAG-mediated signaling in SZ.

In another area, which may be of particular interest, miR-137 was shown to be subject to epigenetic regulation by MeCP2 [61]. MeCP2 and a transcription factor form a complex which binds the miR-137 promoter region to inhibit transcription of miR-137 [76]. miR-137 and MECP2 have been identified as upstream regulators of various genes in our SZ cohort (see Results section Supplementary Table 9 above). Rett syndrome is a neurodevelopmental autism spectrum disorder caused by mutations, which affect germlines in any region of the X-linked MeCP2 gene [77–79]. Two SNPs within the MeCP2 gene have been implicated in SZ [80, 81]. Childhood-onset schizophrenia has been linked with mutations in MECP2 [81–84]. In our study, MeCP2 was identified as an upstream transcription regulator (see Results section S9 above). While there is evidence for the involvement of *de novo* mutations in SZ and autism spectrum disorders [84, 85], a role for MeCP2 targets in SZ has not been shown to date. MeCP2 may regulate region- or cell-specific gene expression and is thought to have a role in the maintenance of dendritic complexity of maturing/mature neurons, maintaining excitatory synapse numbers, and in synaptic scaling of specific neurons [86–89]. The function of MeCP2 in specific brain regions, neuronal populations, and various cell types is becoming more defined, and there is an expression of the MeCP2e2 isoform specific to dorsal thalamus (mouse) [90–92]. If MeCP2 plays a role in the localized regulation of a subset of genes as well as chromatin maintenance in specific cells [93, 94], a disruption of downstream target(s) may also result in dysregulation of activity-dependent dendritic growth and/or synaptic maintenance. The specific target of MeCP2 is not yet identified in RETT to our knowledge. However, ELAV-like protein 1 (ELAVL1; HuR) is one of the many MeCP2 interactants (NCBI). It is important in the regulation and stabilization of the translation of target mRNAs [95, 96]. Previously, two targets of ELAVL1 were identified in SZ [84]. Curiously, manual curation of our data identified an almost 10-fold difference in gene expression for ELAVL1 targets (S9) suggesting possible transcription repression in SZ. Gene expression changes of various ELAVL1 interactants in our study may reflect transcriptional dysregulation of downstream targets of ELAVL1. Our earlier transcriptome study demonstrated SZ-associated gene expression differences in another thalamic region [29]. Of those 31 qPCR validated genes, 8 interact directly with ELAVL1. ELAVL1 has an important role in mRNA stability (AU-rich element mediated) and translation [95, 97]. ELAVL1 is localized to dendrites following experience-dependent synaptic plasticity and may have a role in neuronal mRNA posttranscriptional regulation [98–100]. Posttranslational modification of ELAVL1 may regulate its ability to stabilize mRNA targets [101]. Our findings (S9) lend support to the hypothesis that ELAVL1-related genes (or absence of ELAVL1 target genes) may represent transcription repression and be disease specific for a thalamic region impacted by SZ. While MeCP2 targets have yet to be identified, we hypothesize that ineffective MeCP2 transcription regulation in the SZ cohort may occur via changes in one of its interactant targets, ELAVL1. To our knowledge, no study has identified ELAVL1 with either SZ or MeCP2.

Schizophrenia is a polygenic mental illness, with rare variants of large effect, common variants of small effect, and multiple variants of modest effect are competing for consideration in the genetic complexity of the disorder [102–104]. Our transcriptome findings are related to the previously identified GWAS miRNA-137, other SZ-associated microRNAs, and genes related to ELAVL1, BDNF, DISC-1, and YWHAG associated findings, synapses, and receptors. We hypothesize that our ELAVL1-related findings indicate transcription repression in a specific thalamic nucleus and neural circuit in SZ. To our knowledge, this study is the first to identify miR-137 as an upstream regulator of target genes identified in human postmortem tissues from schizophrenia subjects.

## Figures and Tables

**Table 1:** Demographics. Total number of subjects (*n* = 22), schizophrenia cohort (*n* = 11), and normal control cohort (*n* = 11) for anterior nucleus (AN), and ventral posterior lateral (VPL) comparisons (Supplementary Tables 1–9). Female (F), male (M), and postmortem interval (PMI). From the original cohort (*n* = 15 each, SZ or NC), samples with failed neuron-capture or probe sets with low intensity values or outliers (poor RNA quality or hybridization) were excluded.

Subject	Gender	Duration of disease (years)	Age at death (years)	PMI (hrs)	Used for comparison in Supplementary Table #
SZ	F	8	30	60	1
SZ	M	32	52	61	1, 2, 3
SZ	M	17	30	32	1
SZ	F	24	62	26	1, 2, 3
SZ	F	45	60	40	1, 3
SZ	M	33	60	31	1, 2, 3
SZ	M	5	32	19	1
SZ	M	27	44	50	1, 2, 3
SZ	F	32	56	12	1, 2, 3
SZ	M	16	35	35	1, 2, 3
SZ	F	24	49	38	1, 2, 3
NC	M		52	28	2, 4
NC	M		52	8	1
NC	M		52	22	1, 2, 4
NC	M		53	28	1, 4
NC	M		44	10	1, 2, 4
NC	F		35	23	1, 2, 4
NC	M		41	11	1, 2, 4
NC	M		42	27	1, 2, 4
NC	F		35	40	1
NC	F		68	13	1, 2, 4
NC	F		57	26	1, 2, 4

**Table 2:** Summary of transcripts showing differential expression in each condition.

Comparison	Supplementary Table	Number of differentially expressed transcripts	Hypothetical	Upregulated genes	Downregulated genes
		*P* < 0.05, fold change 2			
SZ AN vs. NC AN	1	528	9	55	482
SZ VPL vs. NC VPL	2	40	6	24	22
		*P* < 0.05 adj.			
SZ AN vs. SZ VPL	3	200	5	24	181
NC AN vs. NC VPL	4	1,984	60	878	1,166

**Table 3:** Schizophrenia-associated microRNAs found across independent studies (reviewed in [55]) identified in our SZ_VPL vs. AN comparison (Supplementary Table 3.3). Their targets, found in our SZ cohort, are bolded.

Upstream regulator in our study	SZ-associated miRNA studies identified the same miRNAs	Target molecules for micro-RNA in our study (from Supplementary Table 3 (S3))
miR-17-5p (and other miRNAs w/seed AAAGUGC)	Santarelli et al. 2011	**ACBD5**, **AHNAK**, **AKAP13**, **ANTXR1**, **APCDD1**, **ATP1A2**, **AUNIP**, **CABLES1**, **CADM2**, **CELF2**, **CFL2**, **CHRM2**, **CMTM4**, **CYBB**, **EFCAB5**, **ETV1**, **FRMD4A**, **FZD3**, **GABRA1**, **GDF11**, **GLIS3**, **IRF9**, **ITPKBLIMA1**, **LRIG1**, **LRP4**, **MAP3K11**, **MAPK4**, **MLL**, **MLL3**, **MTSS1L**, **MYO10**, **NFIB**, **NRP2**, **NTRK3**, **PLCB1**, **POLR3G**, **PPARA**, **QKI**, **RGMA**, **RHOC**, **RYBP**, **SCAMP2**, **SDC2**, **SEMA4B**, **SOBP**, **SPRY4**, **SYT16**, **SYT7**, **TENM1**, **TIMP2**, **TNRC6C**, **TP53INP2**, **TPPP**, **ZNF367**, **ZNF697**, **ZNF704**
miR-151-3p (and other miRNAs w/seed UAGACUG)	Gardiner et al., 2012	**API5**, **CNTN2**, **DSCC1**, **ENTPD7**, **KIAA1217**, **LAPTM5**, **MCM6**, **MYRIP**, **QKI**, **RERG**, **RGS6**, **RYBP**, **RYR3**, **TADA1**

**Table 4:** Synapse-relevant microRNAs (Gerhard [42]) found in our study.

Our data	Synapse related microRNAs identified in [42] (i.e., neuron-specific miR-124)				
Supplementary Table 1.3 AN_SZ-NC	miR-26a-5p miR132-3p	miR-219a-5p miR-219a-1-3p			
Supplementary Table 2.3 VPL_SZ-NC	miR-124-3p miR-132	miR-134-3p miR-219a-1-3p			
Supplementary Table 3.3 AN_VPL-SZ	miR-9-5p miR-26a-5p	miR-134-3p miR-138-5p	miR-132-3p miR-124-3p	miR-219a-5p	
Supplementary Table 4.3 AN_VPL-NC	miR-9 miR-9-5p miR-219a-2-3p	miR-138-5p miR-219a-1-3p miR-219b-5p	miR-134-3p miR-132-3pmiR-219b-3p	miR-124 miR-124-3p miR-219a-5p	miR-26a-5p

## Data Availability

Supplementary Tables will be included with the publication.

## Supplementary Materials

Supplementary Materials Supplementary Table S1: AN disease vs. AN normal control (AN SZ/NC). S1.1: Ingenuity Pathway Analysis—networks AN SZ/NC. S1.2: Ingenuity Pathway Analysis—pathways AN SZ/NC. S1.3: Ingenuity Pathway Analysis—upstream regulators AN SZ/NC. Supplementary Table S2: VPL disease vs. VPL normal control (VPL SZ/NC). S2.1: Ingenuity Pathway Analysis—networks VPL SZ/NC. S2.2: Ingenuity Pathway Analysis—pathways VPL SZ/NC. S2.3: Ingenuity Pathway Analysis—upstream regulators VPL SZ/NC. Supplementary Table S3: VPL and AN disease same subject (SZ_VPL vs. AN). S3.1: Ingenuity Pathway Analysis—networks SZ_VPL vs. AN. S3.2: Ingenuity Pathway Analysis—pathways SZ_VPL vs. AN. S3.3: Ingenuity Pathway Analysis—upstream regulators SZ_VPL vs. AN. S4: VPL and AN normal control same subject NC_VPL vs. AN). S4.1: Ingenuity Pathway Analysis—networks NC_VPL vs. AN. S4.2: Ingenuity Pathway Analysis—pathways NC_VPL vs. AN. S4.3: Ingenuity Pathway Analysis—upstream regulators NC_VPL vs. AN. S5: miR-137 comparisons in our data. S6: our data comparisons to [39] (their Table 1) Potential impact of miR-137 and its targets in schizophrenia. Front Genet Apr 26; 4 : 58. S7: our data comparisons to [40] Inhibition of the schizophrenia-associated microRNA-137 disrupts Nrg1alpha neurodevelopmental signal transduction. Cell Rep July 5; 20(1):1-12. S8: our data comparisons to [41] microRNAs sculpt neuronal communication in a tight balance that is lost in neurological disease Front Mol Neurosci Dec 12; 11 : 455. S9: comparison of ELAVL1 NCBI identified interactants for NC AN/VPL (Table S4) with SZ AN/VPL (Table S3). S10: synapse and receptor related.
